# Robotic-assisted radical prostatectomy with a single port platform: current and future perspectives of a referral center

**DOI:** 10.1590/S1677-5538.IBJU.2022.9978

**Published:** 2022-07-07

**Authors:** Marcio Covas Moschovas, Cathy Corder, Vipul Patel

**Affiliations:** 1 AdventHealth Global Robotics Institute Celebration FL USA AdventHealth Global Robotics Institute, Celebration, FL, USA; 2 University of Central Florida Orlando FL USA University of Central Florida (UCF), Orlando, FL, USA

## COMMENT

Several authors have described the robotic surgery benefits and outcomes in urological procedures, and numerous generations of multiport consoles have been created since the first robotic surgery was approved by the FDA (Food and Drug Administration) in 2000. In this context, all platforms had something in common: the multiport design with several independent arms responsible for controlling the instruments individually.

The minimally invasive surgical concept is always evolving and looking for alternatives to further reduce surgical trauma while improving outcomes. In this scenario, an innovative Single Port (SP) robotic platform was recently cleared by the FDA in urologic procedures. The da Vinci SP is different from the previous robotic generations because instead of independent arms working through multiple abdominal incisions, the SP has only one trocar that houses three biarticulated arms and one flexible scope, allowing the same surgical procedure to be realized with a single access and fewer abdominal incisions (1).

We previously compared the short-term outcomes of da Vinci Xi and SP platforms in patients who underwent radical prostatectomy (2). Despite the increased operative time for the SP group, mainly due to the delicate instruments and learning curve, we could describe less blood loss with minimal intraoperative complications. In our experience, having a less invasive robotic technology facilitated the surgery in patients with renal transplants, inflatable penile prosthesis (IPP), ileostomies, and colostomies. These patients usually have extensive adhesions, increasing the challenges to place multiple trocars. In addition, these cases have intraabdominal obstacles (ileostomy, colostomy, prosthesis reservoir, and pelvic transplanted kidney), which blocks the optimal triangulation and working space of multiport consoles. Therefore, placing the SP trocar on the midline facilitates the appropriate instrument deployment without limiting the movements on the sides, decreasing the chances of clashing, and damaging these internal structures.

Furthermore, in our experience, the SP platform is a less invasive option for lymphocele drainage in symptomatic patients with previous Interventional Radiology (IR) drainage attempts (3). Instead of six conventional incisions done with the multiport robot, the same surgical drainage is performed with only one or two abdominal accesses, which enables definitive treatment of the lymphocele sac and fewer days with abdominal drain.

As an oncological referral center, we believe that treatment delays affect the outcomes of each patient, and the appropriate therapy must be provided as soon as possible to optimize results. Patients with the abovementioned circumstances usually have their surgical treatment deferred or even canceled (sent to radiation) due to potential challenges faced during surgery, such as bowel injury, prolonged operative time, and even aborted procedures when the multiport trocar placement is not feasible. Therefore, we believe that the current SP generation is not a replacement of the multiport but an option for these patients to avoid treatment delays, providing appropriate oncological management through only one or two abdominal incisions (SP plus the assistant trocar).

**Figure 1:**
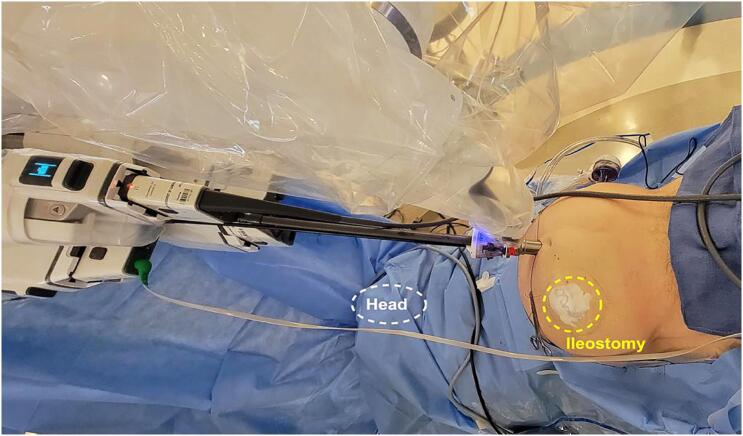
Da Vinci SP radical prostatectomy in a patient with ileostomy and rectal amputation.

We recently described our experience in patients undergoing radical prostatectomy with the SP robot, and we found that by maintaining selection criteria, using only two ports (instead of six), the trends of positive surgical margins have minimal variations during the learning curve, with rates compatible with our multiport approach (4). In addition, our experience performing lymphocele drainage is positive due to the less invasive procedure on these patients, allowing for definitive treatment with a single incision (3). Finally, the SP robot benefits patients with ileostomy, colostomy, prosthesis reservoir, and transplanted kidney with satisfactory intraoperative outcomes, minimizing delays and complications on the definitive treatment. Figure-1 illustrates the SP docked in a patient with rectal amputation and ileostomy (SP trocar supraumbilical and midline, and 12mm assistant trocar on the left lower quadrant). The right side was totally blocked by the ileostomy and bowel adhesions, which impeded the multiport trocar placement.

The initial barrier to the SP implementation is the increased price of the robotic platform, instruments, and disposables compared to the multiport (5). However, by adopting same-day discharge protocols in selected cases and minimizing the hospitalization time, some centers could reduce the final surgical cost. The second barrier is that this platform is available only in a few centers (USA, Korea, and Hong Kong), and only some specialties are cleared by the FDA to use it in clinical settings. Finally, this platform demands a new learning curve and training requirements for the whole team, which may not motivate expert surgeons established for years with the multiport robot.

Single Port surgery, as previously described, provides a less invasive robotic approach with potential benefits to some patients that are not candidates for the multiport. However, the current literature is based on retrospective studies with its inherent risks of bias. Most studies have less than 150 patients with short-term outcomes (6). We believe that the SP technology diffusion depends on well-designed comparative studies performed by referral centers, reporting long-term outcomes. In this scenario, considering the higher costs and the new learning process associated with this robot, some institutions maintain the multiport console while waiting for mature data to justify the SP investment.

Since the SP clearance by the FDA, we observed an expansion of this technology in academic centers, which may benefit the diffusion of this robot in the following years due to the early exposure of residents, fellows, and surgeons training robotics. In addition, the experience of these centers in terms of reducing costs, optimizing outcomes, and describing their experience with the technological implementation, enables smaller centers to minimize initial mistakes and complications during the learning curve, which are also factors to influence the diffusion of this technology.

Finally, considering that the current SP robot is the first version of this technology, we believe that Single Port surgery will be the standard approach with succeeding generations and its expansion will gain traction with long-term outcomes reports, decreased costs, and instruments improvements.

## References

[1] Covas Moschovas M, Bhat S, Rogers T, Onol F, Roof S, Mazzone E, et al. Technical Modifications Necessary to Implement the da Vinci Single-port Robotic System. Eur Urol. 2020;78:415-23. 10.1016/j.eururo.2020.01.005 31959548

[2] Moschovas MC, Bhat S, Sandri M, Rogers T, Onol F, Mazzone E, et al. Comparing the Approach to Radical Prostatectomy Using the Multiport da Vinci Xi and da Vinci SP Robots: A Propensity Score Analysis of Perioperative Outcomes. Eur Urol. 2021;79:393-404. 10.1016/j.eururo.2020.11.042 33357994

[3] Reddy S, Moschovas MC, Bhat S, Noel J, Helman T, Perera R, et al. Minimally invasive lymphocele drainage using the Da Vinci® single port platform: step-by- step technique. Int Braz J Urol. 2022;48:363-4. 10.1590/S1677-5538.IBJU.2021.0272 PMC8932025 35170903

[4] Covas Moschovas M, Kind S, Bhat S, Noel J, Sandri M, Rogers T, et al. Implementing the da Vinci SP Without Increasing Positive Surgical Margins: Experience and Pathologic Outcomes of a Prostate Cancer Referral Center. J Endourol. 2022;36:493-8. 10.1089/end.2021.0656 34963334

[5] Moschovas MC, Helman T, Bhat S, Sandri M, Rogers T, Noel J, et al. Does type of robotic platform make a difference in the final cost of robotic-assisted radical prostatectomy? J Robot Surg. 2022;28. Epub ahead of print. Erratum in: J Robot Surg. 2022 Feb 25; 10.1007/s11701-021-01359-5 35089500

[6] Covas Moschovas M, Bhat S, Rogers T, Thiel D, Onol F, et al. Applications of the da Vinci single port (SP) robotic platform in urology: a systematic literature review. Minerva Urol Nephrol. 2021;73:6-16. 10.23736/S2724-6051.20.03899-0 32993277

